# Microsatellite and *Wolbachia* analysis in *Rhagoletis cerasi* natural populations: population structuring and multiple infections

**DOI:** 10.1002/ece3.553

**Published:** 2014-04-21

**Authors:** Antonios A Augustinos, Anastasia K Asimakopoulou, Cleopatra A Moraiti, Penelope Mavragani-Tsipidou, Nikolaos T Papadopoulos, Kostas Bourtzis

**Affiliations:** 1 Department of Environmental and Natural Resources Management, University of Western GreeceAgrinio, Greece; 2 Department of Biochemistry and Biotechnology, University of ThessalyLarissa, Greece; 3 Insect Pest Control Laboratory, Joint FAO/IAEA Programme of Nuclear Techniques in Food and AgricultureVienna, Austria; 4 Department of Agriculture, Crop Production and Rural Environment, University of ThessalyN. Ionia (Volos), Magnesia, Greece; 5 Department of Genetics, Development and Molecular Biology, School of Biology, Faculty of Sciences, Aristotle University of ThessalonikiThessaloniki, Greece; 6 Biomedical Sciences Research Center Al. FlemingVari, Greece

**Keywords:** Insect symbiosis, microsatellites, *Rhagoletis cerasi*, Tephritidae, *Wolbachia*

## Abstract

*Rhagoletis cerasi* (Diptera: Tephritidae) is a major pest of sweet and sour cherries in Europe and parts of Asia. Despite its economic significance, there is a lack of studies on the genetic structure of *R. cerasi* populations. Elucidating the genetic structure of insects of economic importance is crucial for developing phenological-predictive models and environmental friendly control methods. All natural populations of *R. cerasi* have been found to harbor the endosymbiont *Wolbachia pipientis,* which widely affects multiple biological traits contributing to the evolution of its hosts, and has been suggested as a tool for the biological control of insect pests and disease vectors. In the current study, the analysis of 18 *R. cerasi* populations collected in Greece, Germany, and Russia using 13 microsatellite markers revealed structuring of *R. cerasi* natural populations, even at close geographic range. We also analyzed the *Wolbachia* infection status of these populations using 16S *rRNA*-, MLST- and *wsp*-based approaches. All 244 individuals screened were positive for *Wolbachia*. Our results suggest the fixation of the *w*Cer1 strain in Greece while *w*Cer2, *w*Cer4, *w*Cer5, and probably other uncharacterized strains were also detected in multiply infected individuals. The role of *Wolbachia* and its potential extended phenotypes needs a thorough investigation in *R. cerasi*. Our data suggest an involvement of this symbiont in the observed restriction in the gene flow in addition to a number of different ecological factors.

## Introduction

**Figure 1:**
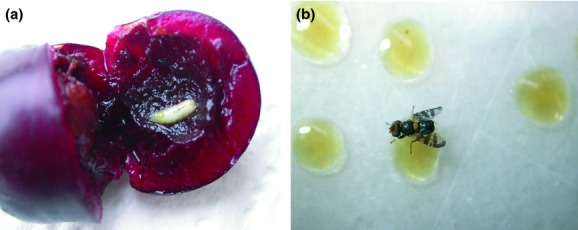
*Rhagoletis cerasi* fly: (a) third instar larvae of *R. cerasi* infesting a cherry fruit and (b) *R. cerasi* adult (male) on food droplets.

The European cherry fruit fly, *Rhagoletis cerasi* L. (Diptera: Tephritidae) (Fig. 1)*,* is a pest of major agricultural importance infesting mainly sweet (*Prunus avium*) and sour (*P. cerasus*) cherries as well as honeysuckle (mainly *Lonicera tatarica* and *L. xylosteum*) and wild growing prunus. *Rhagoletis cerasi* has dispersed and become established in almost all European countries, spreading from the original habitat, the Caucasian area of western Asia (Fimiani 1989; White and Elson-Harris 1992). *Rhagoletis cerasi* follows a univoltine life cycle with obligatory pupa dormancy that lasts over 9 months. Adults emerge in spring and oviposit usually a single egg on ripe or ripening fruits (Fletcher 1989; Daniel and Grunder 2012). Larvae feed on mesocarp, destroying fruits and causing considerable economic loss exacerbated by secondary fungal and bacterial infections (Fimiani 1989). Because of the economic importance, the control of the European cherry fruit fly received much attention in the framework of the sterile insect technique (SIT) in the early 1970s (Boller et al. 1971, 1977; Boller and Katsoyannos 1977).

A number of studies have focused on the ecology of *R. cerasi* (Boller and Prokopy 1976; Boller et al. 1998; Kovanci and Kovanci 2006; Papanastasiou et al. 2011; Moraiti et al. 2012a,b2012b) or the development of environmental friendly control methods, such as mass trapping, application of the oviposition deterring pheromone, bait sprays using plant-derived insecticides and biological control (Katsoyannos et al. 2000; Daniel and Grunder 2012). However, very little is known regarding the biology of this pest at the molecular, genetic, and population levels. The cytogenetics (including the development of polytene chromosome maps) have been recently studied (Kounatidis et al. 2008), though the genetic structure of the European cherry fruit fly populations has received much less attention. Allozyme polymorphisms (Schwarz et al. 2003) raise the question of host race formation in this species, due to host shift, and a small-scale analysis based on microsatellite markers (Augustinos et al. 2011a) points toward elevated genetic differences even in a short geographical range.

In fruit flies (other than *Rhagoletis* species), microsatellites have been used to shed light on: recent invasion events and dispersion routes of several invasive species (Meixner et al. 2002; Bonizzoni et al. 2004; Augustinos et al. 2005; Nardi et al. 2005; Aketarawong et al. 2007; Khamis et al. 2009; Zygouridis et al. 2009; Virgilio et al. 2010); the degree of genetic structuring in natural populations that can be associated with incipient speciation (Michel et al. 2007); and pattern of remating in nature (Bonizzoni et al. 2002; Song et al. 2007). Lately, microsatellite markers have been also used to address phylogenetic issues in the *Bactrocera musae* complex of species (Drew et al. 2011).

Microsatellite markers have been recently developed for *R. pomonella* (Velez et al. 2006), *R. indifferens* (Maxwell et al. 2009), and *R. cerasi* (Arthofer et al. 2009a) and used to: (a) address issues regarding speciation events in *R. pomonella* (Michel et al. 2007, 2010; Forbes et al. 2009), and (b) analyze native and introduced populations of *R. completa* (Chen et al. 2010). The recent development of 13 microsatellite markers for *R. cerasi* (Arthofer et al. 2009a), along with the evaluation of cross-amplified microsatellite markers from other *Rhagoletis* species (Augustinos et al. 2011a), provide a solid basis for exploring the population genetics of this species.

*Wolbachia pipientis* (*Wolbachia* for the purpose of this manuscript) is perhaps the most widespread and abundant symbiont among insect species. Extensive genetic diversity has been recorded in the *Wolbachia* group with the different strains assigned in 13 supergroups, namely A to F and H to N (Augustinos et al. 2011b). The genotyping of the *Wolbachia* strains is based on either single gene (such as *wsp*, *ftsZ*, *gltA*, *groEL*, *dnaA*) or Multi Locus Sequence Typing (MLST) approaches (Casiraghi et al. 2005; Baldo et al. 2006; Paraskevopoulos et al. 2006).

The widespread distribution of *Wolbachia*, its ability to manipulate reproductive properties, as well as other important physiological functions of its arthropod hosts, have attracted the attention of investigators regarding the role of this symbiont in host biology, ecology, and evolution (Serbus et al. 2008; Werren et al. 2008; Saridaki and Bourtzis 2010). In addition, *Wolbachia* symbiosis is currently being explored and harnessed toward population control of insect pests and disease vectors (Zabalou et al. 2004, 2009; Brelsfoard and Dobson 2009; Apostolaki et al. 2011; Atyame et al. 2011; Walker et al. 2011).

Revealing the close association between *R. cerasi* and *Wolbachia,* Riegler and Stauffer shed important light on the reproductive incompatibility reported in the mid-1970s between northwest and southeast European populations of this pest (Boller et al. 1976; Matolin 1976; Riegler and Stauffer 2002). So far, five *Wolbachia* strains of *R. cerasi* have been described (*w*Cer1 to *w*Cer5). The identification of the first two strains, *w*Cer1 and *w*Cer2, was based mainly on *wsp* gene divergence. Wild *R. cerasi* populations were found to be infected either with *w*Cer1, or super infected with *w*Cer1 and *w*Cer2. Moreover, *w*Cer2 was proposed to be the causal factor for the reproductive incompatibility of the aforementioned populations, now known as *Wolbachia*-induced cytoplasmic incompatibility (CI) (Riegler and Stauffer 2002). Based on a polymerase chain reaction (PCR) screen with *Wolbachia-*specific primers for 16S *rRNA* and *wsp,* Kounatidis et al. (2008) reported that the four Greek populations tested were also 100% infected. The *Wolbachia* strains *w*Cer3, *w*Cer4, and *w*Cer5 were only recently isolated from *R. cerasi* populations (Arthofer et al. 2009b, 2011). Interestingly, *w*Cer4 was identified only after *Ceratitis capitata* transinfection with *Wolbachia,* using *R. cerasi* as donor (Zabalou et al. 2004). Arthofer et al. (2011) proposed Allelic Intersection Analysis as a novel tool for the MLST assignment of multiply infected hosts and described the MLST profile of *R. cerasi Wolbachia* strains *w*Cer1 to *w*Cer5 (except *w*Cer3), indicating that *w*Cer1*, w*Cer2, *and w*Cer4 belong to *Wolbachia* supergroup A, while *w*Cer5 belongs to supergroup B. The *Wolbachia* strain *w*Cer3 is supposed to be a recombinant A/B strain, according to the available *wsp* gene sequence data (Arthofer et al. 2009b).

Recent studies showed that *R. pomonella* (Schuler et al. 2011) and *R. cingulata* (Drosopoulou et al. 2011) are infected by *Wolbachia,* while *R. completa* seems to be free of *Wolbachia* (Drosopoulou et al. 2010). *Rhagoletis pomonella* is infected with the *w*Pom strain, which seems to be very similar (or identical) to *w*Cer2, while other sequence variants for different MLST genes (not attributed to specific strains) have also been reported (Schuler et al. 2011). *Rhagoletis cingulata* is infected with at least two strains very similar (or identical) to *w*Cer1 and *w*Cer2 (Schuler et al. 2009; Drosopoulou et al. 2011), named *w*Cin1 and 2 (Schuler et al. 2009).

*Wolbachia* strains derived from *R. cerasi* have been recently used for the development of an alternative and environment-friendly strategy to control two major agricultural pests: the Mediterranean fruit fly, *C. capitata* (Zabalou et al. 2004, 2009) and the olive fly, *Bactrocera oleae* (Apostolaki et al. 2011). This technique, the Incompatible Insect Technique (IIT), is in principle analogous to the SIT. The main difference between them is the sterilization factor; the IIT is based on *Wolbachia*-induced CI while the SIT is based on irradiation (Bourtzis and Robinson 2006). IIT holds great potential for the control of agricultural pests and disease vectors (Zabalou et al. 2004; Brelsfoard and Dobson 2009; Saridaki and Bourtzis 2010; Apostolaki et al. 2011; Atyame et al. 2011).

Because of its wide geographical distribution, univoltine life history, patchy distribution of its hosts, low adult dispersion abilities, and infection with different *Wolbachia* strains (Phillips and Dirks 1933; Jones and Wallace 1955; Boller and Prokopy 1976; Fletcher 1989; Kneifl et al. 1997), *R. cerasi* bears several properties of becoming an important model for addressing issues regarding effect of ecological factors and symbionts on the differentiation of genetic, demographic, and behavioral traits (Augustinos et al. 2011a; Moraiti et al. 2012b).

## Materials and Methods

### Collection of fly samples and DNA extraction

**Figure 2:**
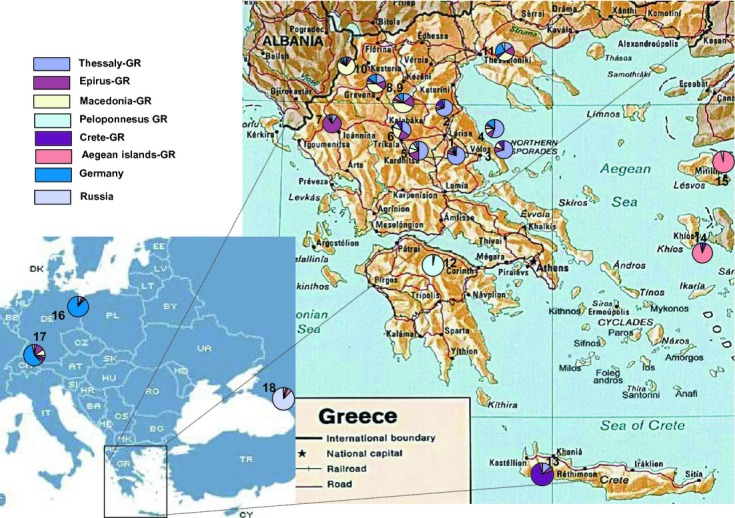
*Rhagoletis cerasi* sampling areas and GeneClass analysis. Sampling sites: 1, Agia Larissa; 2, Kallipefki Larissa; 3, Kato Lechonia; 4, Kamari; 5, Karditsa; 6, Pertouli; 7, Konitsa; 8, Dafni1; 9, Dafni2; 10, Kastoria; 11, Salonica; 12, Kernitsa; 13, Chania; 14, Chios; 15, Lesvos; 16, Steckelberg; 17, Dossenheim; 18, Krasnodar. Samples were grouped for GeneClass analysis according to geographic origin.

A total of 465 adults from 18 different *R. cerasi* popualtions were genotyped using 13 microsatellite markers. Fifteen of the populations came from Greece (12 mainland and 3 island) and additionally three from Germany (two populations) and Russia. In all cases, field-infested sweet cherries were sampled and kept in the laboratory until the collection of pupae. Adults emerging from pupae were stored at −80°C in 95% ethanol. Genomic DNA was extracted as described in Augustinos et al. (2011a). Collection sites and the number of flies used in the study are shown in Figure 2 and Table 1.

**Table 1:** Description of *Rhagoletis cerasi* populations and measure of genetic variability

No	Sample	Region	Country	Collection date	Longitude	Latitude	Plant host	*N*	*n*_a_	*n*_e_	AR	*H*_o_	*H*_e_	HWE
1	Agia-GR	Thessaly	Greece	2008	39°43′04″N	22°45′49″E	Sweet cherry	30	4.33	2.14	3.74	0.47	0.47	1/13
2	Kallipefki-GR	Thessaly		2008	39°58′0″N	22°27′37″E	Sweet cherry	20	3.58	2.02	3.42	0.41	0.44	–
3	Kato Lechonia-GR	Thessaly		2008	39°19′49″N	23°02′17″E	Sweet cherry	18	3.08	2.09	3.06	0.37	0.45	3/13
4	Kamari Pilio-GR	Thessaly		2008	39°34′47″N	22°55′5.40″E	Wild sweet cherry	20	3.33	2.01	3.24	0.39	0.41	–
5	Karditsa-GR	Thessaly		2008	39°36′N	21°92′E	Sweet cherry	20	3.25	2.02	3.16	0.37	0.44	2/13
6	Pertouli-GR	Thessaly		2008	39°32′19″N	21°27′58″E	Wild sweet cherry	20	3.50	2.14	3.42	0.42	0.46	1/13
7	Konitsa-GR	Epirus		2008	40°2′44″N	20°44′52″E	Sweet cherry	30	4.00	2.02	3.60	0.37	0.43	2/13
8	Dafni1-GR	Macedonia		2008	40°17′08″N	21°08′53″E	Sweet cherry	20	3.67	1.90	3.46	0.36	0.42	1/13
9	Dafni2-GR	Macedonia		2008	40°17′08″N	21°08′53″E	Prunus sp.	28	3.75	2.03	3.35	0.40	0.46	1/13
10	Kastoria-GR	Macedonia		2008	40°31′34″N	21°15′47″E	Sweet cherry	30	4.08	2.07	3.65	0.40	0.45	1/13
11	Thessaloniki-GR	Macedonia		2008	40°38′19″N	22°56′43″E	Sweet cherry	19	3.83	2.19	3.74	0.44	0.48	2/13
12	Kernitsa-GR	Peloponnesus		2008	38°7′60″N	22°13′0″E	Sweet cherry	30	3.00	1.94	2.89	0.36	0.42	2/13
13	Chania-GR	Crete		2008	35°51′N	24°01′E	Sweet cherry	30	3.42	1.98	3.19	0.41	0.47	–
14	Chios-GR	East Aegean		2008	38°21′06″N	26°08′30″E	Sweet cherry	30	2.92	2.00	2.81	0.35	0.42	3/13
15	Lesvos-GR	East Aegean		2008	39°06′13″N	26°31′59″E	Sweet cherry	30	3.00	2.14	2.79	0.39	0.44	1/13
16	Stecklenberg-GER		Germany	2008	51°73′0″N	11°08′E	Sweet cherry	30	4.17	2.10	3.61	0.41	0.45	–
17	Dossenheim-GER			2008	49°27′0″N	8°40′0″E	Sweet cherry	30	4.08	2.01	3.56	0.39	0.40	–
18	Krasnodar-RUS		Russia	2008	45°2′0″N	38°58′0″E	Uknown host	30	4.33	2.51	3.91	0.48	0.52	3/13
	Mean							25.8	3.63	2.07	3.36	0.40	0.45	

*N*, sample size; *n*_a_, number of actual alleles; *n*_e_, number of effective alleles; AR, allelic richness; *H*_o_, observed heterozygosity; *H*_e_, expected heterozygosity; HWE, loci that deviate from equilibrium, according to G^2^ criterion, at a significance level of 0.05.

### Microsatellite genotyping and data analysis

PCRs, genotyping, and specific allele sequencing of microsatellite markers were performed as described in Augustinos et al. (2011a). PCR conditions for markers Rce76-1 and 83-44 were similar to those described in Arthofer et al. (2009a).

Genetic variability was measured as the mean number of alleles per locus (*n*_a_), effective number of alleles (*n*_e_), observed (*H*_o_) and expected heterozygosity (*H*_e_), using POPGENE, version 1.31 (Yeh et al. 1999). Allelic diversity was determined after correction for sample size, using FSTAT (Goudet 2001). Deviations from Hardy–Weinberg Equilibrium (HWE) were tested with the G^2^ likelihood ratio test in POPGENE. Genotypic disequilibrium was tested with Genepop (Raymond and Rousset 1995), using Fisher's exact test, for all pairs of loci in all populations and across them. Presence of null alleles was tested with ML-Null software. Hardy–Weinberg tests for heterozygote deficiency were performed (Rousset and Raymond 1995), after performing 10,000 randomization tests using MonteCarlo randomization as described by Guo and Thomson (1992). Genetic distances were measured according to Nei (1972), using POPGENE (Yeh et al. 1999). Population differentiation was estimated using the FSTAT software, using a significance level of 0.05, adjusted for multiple comparisons. Software GENALEX 6.5 (Peakall and Smouse 2006) was used for the estimation of the pairwise population PhiPT values. This PhiPT matrix was used in Genalex 6.5 to perform Principal Components Analysis (PCA). PHYLIP 3.6c (Felsenstein 1994) was used for the construction of an unrooted UPGMA dendrogram after 1000 bootstrap resamples, using allele frequencies, as described in Augustinos et al. (2005). STRUCTURE v2.2 software was used to determine the number of possible genetic clusters (Pritchard et al. 2000). We used all four different models implemented in this program, assuming the presence of admixture and without admixture, for independent or correlated allele frequencies, with a burn-in period of 50,000 and 500,000 Marcov Chain Monte Carlo (MCMC) repetitions after the initial burn-in. We tested for *K* = 1 to *K* = 12 (where *K* stands for the assumed number of populations). We also used the modification described by Evanno et al. (2005) to predict the true number of clusters in complex situations more accurately. For this purpose, we used the “no admixture” model with correlated frequencies, with a burn-in period of 50,000 and 500,000 MCMC repetitions after the initial burn-in. We ran 10 repetitions of the above model, assuming *K* = 1 to *K* = 12. We chose the specific model because, as it is suggested by the authors of the program, it can better resolve “complicated” or “weak” cases of structuring. Software GeneClass 2.0 (Piry et al. 2004) was used to perform population assignment and exclusion test and to calculate the probability of origin for each individual and each population, under a Bayesian model using the Rannala and Mountain (1997) criterion with a 0.05 threshold. Isolation by distance was tested using the “Mantelize it” option of the FSTAT software (Goudet 2001) for plotting genetic distances against geographic distances, and Pearson's *r* coefficient was measured using Excel. Finally, the BOTTLENECK software was used (Cornuet and Luikart 1996) to detect any recent bottleneck phenomena. This software runs under the assumption that recent bottlenecks lead to a shift from the L-shaped distribution of allele frequencies along with a quicker loss of rare alleles than heterozygosity. However, the low number of microsatellite loci used, especially if not highly polymorphic, can lead in reduced “ability” to detect bottleneck phenomena. In the frame of this study, the Mode shift and the Wilcoxon sign rank tests implemented in this software were used.

### *Wolbachia* genotyping and data analysis

*Wolbachia* infections from six individual flies belonging to separate genetic clusters (according to microsatellite analysis) and/or of different geographic origin were genotyped by sequencing of the 16S *rRNA*, *wsp,* and MLST genes. A 16S *rRNA* gene fragment (438 base pairs) was amplified with the *Wolbachia*-specific primers *wsp*ecF and *wsp*ecR (Werren and Windsor 2000). Gene fragments of *wsp* and the MLST genes (*gatB, coxA, hcpA, fbpA,* and *ftsZ*) were amplified using the respective primers and conditions reported previously (Casiraghi et al. 2005; Baldo et al. 2006; Ros et al. 2009).

### Cloning and sequencing

After detecting multiple peaks in preliminary sequencing reactions, PCR products of all genes were cloned, sequenced, and assembled as described in Augustinos et al. (2011b). Three to six colonies were directly subjected to PCR, using the primers T7 and SP6 and double-strand sequenced.

### Phylogenetic analysis

All *Wolbachia* gene sequences generated were aligned using the ClustalW algorithm (Thompson et al. 1994), implemented in MEGA (Tamura et al. 2011). Sequences obtained from GenBank representing all currently known supergroups of *Wolbachia* were included in the analysis. Phylogenetic reconstruction was performed using the Maximum Likelihood (ML) method, after 500 bootstrap resamples of the original data.

### Recombination analysis

Sequences for all genes (MLST, *wsp,* 16S *rRNA*) were tested for recombination events using the RDP3 package (Martin et al. 2010). All the available modules (RDP3, BOOTSCAN, GENECONV, MAXCHI, CHIMAERA, SISCAN, 3SEQ, VisRD, LARD, and LDHAT) were implemented under the default options. As the authors of the software state, the “default options” are sufficient to detect recombination events for most of the datasets even without the need of reference nonrecombinant sequences.

### Screening for *w*Cer specific strains

Ten to twenty flies per population were screened for the presence of *w*Cer1 to *w*Cer5 *Wolbachia* strains using a *wsp* gene-based PCR assay as previously reported (Arthofer et al. 2009b).

### Nucleotide sequence accession numbers

All microsatellite and *Wolbachia* gene sequences generated in this study have been deposited into GenBank under accession numbers JX870457-JX870517 and as well as to the *Wolbachia* MLST database.

## Results

### Microsatellite marker variability and HWE

The markers used have on average 6.23 *n*_a_ with the mean *n*_e_ being 2.16 (Table 2). The mean observed heterozygosity was lower than the expected (0.39 vs. 0.47). Out of the 234 locus-sample tests performed for HWE, 25 showed deviation according to the G^2^ criterion at a significance level of 0.05. Most of these deviations are concentrated in marker *Rp11* (six) and can be partially attributed to the presence of null alleles, as this locus exhibits an excess of different classes of homozygotes. Analysis performed with the ML-Null software points to the presence of null alleles for this locus in some of the populations. No evidence for extensive presence of null alleles for the other loci was found.

**Table 2:** Genetic variability of microsatellite markers

Marker	Reference	Size variation	*N*	*n*_a_	*n*_e_	AR	*H*_o_	*H*_e_	HWE
RcMic(Boms3b)	Augustinos et al. (2008)	94–136	462	4	1.34	3.33	0.24	0.26	2/18
Rc(Rp1)	Velez et al. (2006) Augustinos et al. (2011)	486–496	433	3	1.88	2.37	0.26	0.47	2/18
RcMic(Rp2)	Velez et al. (2006)	202–220	456	9	2.96	5.40	0.63	0.66	1/18
RcMic(Rp11)	Velez et al. (2006)	289–299	416	5	2.60	3.69	0.40	0.62	6/18
RcMic(Rp12)	Velez et al. (2006)	190–204	463	7	2.47	3.92	0.50	0.60	4/18
RcMic(Rp15)	Velez et al. (2006)	234–252	451	6	1.90	3.29	0.43	0.48	0/18
RcMic(Ri48)	Maxwell et al. (2009)	128–156	454	8	1.50	4.38	0.22	0.33	1/18
RcMic(Ri67)	Maxwell et al. (2009)	180–196	445	6	1.42	3.41	0.27	0.30	0/18
RcMic(Ri83)	Maxwell et al. (2009)	166–170	461	2	1.46	2.00	0.30	0.32	2/18
RcMic(Ri93)	Maxwell et al. (2009)	185–205	449	9	3.70	4.95	0.57	0.73	3/18
RcMic(Ri111)	Maxwell et al. (2009)	204–210	454	3	1.23	2.59	0.14	0.19	4/18
Rce76-1	Arthofer et al. (2009a)	191–213	459	11	3.54	5.1	0.68	0.72	0/18
Rce83-44	Arthofer et al. (2009a)	189–213	459	8	2.00	4.57	0.40	0.50	0/18
Mean			452.5	6.23	2.16	2.371	0.47	0.39	

*N*, sample size; *n*_a_, number of actual alleles; *n*_e_, number of effective alleles; AR, allelic richness; *H*_o_, observed heterozygosity; *H*_e_, expected heterozygosity; HWE, number of samples in which locus deviates from equilibrium, according to G^2^ criterion, at a significance level of 0.05.

### Population polymorphism and HWE

Populations studied exhibit a varying degree of polymorphism with 1.9 to 2.51 *n*_e_ per locus (Dafni1-GR and Krasnodar-RU, respectively). According to all measures, Agia Larissa-GR and Krasnodar-RU are the most polymorphic populations while Kernitsa-GR and the two neighboring islands of Chios-GR and Lesvos-GR present the lowest variability. Sporadic HWE deviations (up to three loci) were observed in few of the populations studied (Table 1).

### Genetic distances and population differentiation

Pairwise genetic distances (Nei 1972) among populations were determined ranging from 0.0140 to 0.1630 (Table 3). *F*_ST_ (fixation index) values were determined and tested at a significance level of 0.05 (Table 4). There are no significant differences among populations from Thessaly, except the one observed between Kamari Pilio-GR/Pertouli-GR. The four populations from Macedonia are also quite homogeneous, with the exception of the difference between Kastoria and Dafni2. The single population from Epirus (Konitsa-GR) shows differentiation with some Thessaly/Macedonia populations and at the same time, it is not significantly differentiated from the Dossenheim population. The populations from Peloponnesus (Kernitsa-GR), Crete (Chania-GR), Aegean islands (Lesvos-GR and Chios-GR), and Russia (Krasnodar-RU) are all differentiated from all previously reported and among them. The German populations seem to form a homogeneous cluster.

**Table 3:** Genetic distances^1^ (above the diagonal) and geographic distances in Kilometers^2^ (below the diagonal) among *Rhagoletis cerasi* populations

	Greece															Germany		Russia
	
	Thessaly						Epirus	Macedonia				Peloponnesus	Crete	Eastern Aegean islands				
								
	1	2	3	4	5	6	7	8	9	10	11	12	13	14	15	16	17	18
Agia Larisa-GR	–	0.0295	0.0365	0.0381	0.0290	0.0416	0.0323	0.0361	0.0388	0.1002	0.0532	0.1017	0.0885	0.1544	0.1061	0.0934	0.0828	0.1526
Kallipef Lar-GR	38.9	–	0.0384	0.0232	0.0366	0.0481	0.0603	0.0581	0.0505	0.1051	0.0562	0.1060	0.0810	0.1220	0.1084	0.0737	0.0595	0.1253
Kato Lehonia-GR	50	87	–	0.0337	0.0584	0.0431	0.0633	0.0443	0.0610	0.0893	0.0612	0.1128	0.0813	0.1580	0.1179	0.0912	0.0819	0.1405
Kamari Pilio-GR	19	49	29	–	0.0393	0.0508	0.0712	0.0444	0.0464	0.0846	0.0441	0.0754	0.0853	0.1170	0.1134	0.0661	0.0525	0.1281
Karditsa-GR	97	85	97	104	–	0.0293	0.0301	0.0309	0.0256	0.0652	0.0340	0.0526	0.0717	0.1131	0.1187	0.0719	0.0560	0.1239
Pertouli-GR	113	93	138	124	33	–	0.0260	0.0294	0.0304	0.0478	0.0257	0.0689	0.0597	0.1314	0.1182	0.0642	0.0418	0.1099
Konitsa-GR	175	147	212	192	116	82	–	0.0299	0.0320	0.0764	0.0338	0.0988	0.0854	0.1496	0.1268	0.0895	0.0657	0.1588
Dafni1-GR	137	107	181	156	110	81	53	–	0.0140	0.0273	0.0213	0.0671	0.0493	0.1076	0.1050	0.0516	0.0410	0.1145
Dafni2-GR	137	107	181	156	110	81	53	0	–	0.0416	0.0211	0.0660	0.0555	0.0824	0.0998	0.0412	0.0326	0.0992
Kastoria-GR	155	125	202	174	137	109	53	28	28	–	0.0315	0.0653	0.0450	0.0954	0.1268	0.0631	0.0435	0.0864
Thessaloniki-GR	103	95	133	117	177	175	67	144	144	142	–	0.0629	0.0488	0.0737	0.0798	0.0464	0.0247	0.0898
Kernitsa-GR	182	193	152	172	139	169	247	249	249	277	285	–	0.0768	0.1201	0.1630	0.1165	0.0870	0.1300
Chania-GR	479	502	432	462	486	500	579	578	578	605	576	332	–	0.0809	0.1111	0.0663	0.0444	0.0840
Chios-GR	328	358	288	309	395	425	500	465	465	482	372	344	368	–	0.0526	0.0828	0.0598	0.0763
Lesvos-GR	306	341	300	298	396	421	488	447	447	458	332	376	454	90	–	0.1186	0.1010	0.0841
Stecklenberg-GER	1611	1584	1568	1630	1606	1574	1497	1497	1497	1471	1531	1743	2075	1892	1829	–	0.0159	0.0986
Dossenheim -GER	1551	1522	1599	1571	1528	1495	1413	1424	1424	1400	1486	1658	1989	1858	1806	305	–	0.0879
Krasnodar-RUS	1456	1471	1453	1452	1553	1563	1591	1538	1538	1530	1395	1589	1649	1297	1230	2180	2329	–

Numbers in third row stand for different samples: 1, Agia Larissa; 2, Kallipefki Larissa; 3, Kato Lechonia; 4, Kamari; 5, Karditsa; 6, Pertouli; 7, Konitsa; 8, Dafni1; 9, Dafni2; 10, Kastoria; 11, Salonica; 12, Kernitsa; 13, Chania; 14, Chios; 15, Lesvos; 16, Steckelberg; 17, Dossenheim; 18, Krasnodar.

Gray shading marks different geographic groups.

1 Genetic distances according to Nei (1972).

2 Geographic distances are in kilometers. Chios, Lesvos and Chania (Crete) are samples from islands. See also Figure 1 for a geographic map of the collection sites and Table 1for precise location.

**Table 4:** Pair-wise test of differentiation (above diagonal) and significance of differentiation, at the 0.05 significance level (below diagonal)

	Greece															Germany		Russia
	
	Thessaly						Epirus	Macedonia-GR				Peloponnesus	Crete	Eastern Aegean islands				
								
	1	2	3	4	5	6	7	8	9	10	11	12	13	14	15	16	17	18
1	–	0.36536	0.26699	0.00229	0.03529	0.00817	0.00033	0.03627	0.00163	0.00033	0.00229	0.00033	0.00033	0.00033	0.00033	0.00033	0.00033	0.00033
2	NS	–	0.14739	0.01667	0.02484	0.01928	0.00098	0.00392	0.00065	0.00033	0.00098	0.00033	0.00033	0.00033	0.00033	0.00033	0.00033	0.00033
3	NS	NS	–	0.01405	0.00098	0.00752	0.00131	0.10229	0.00327	0.00033	0.00131	0.00033	0.00033	0.00033	0.00033	0.00033	0.00033	0.00033
4	NS	NS	NS	–	0.00065	0.00033	0.00033	0.00131	0.00065	0.00033	0.00065	0.00033	0.00033	0.00033	0.00033	0.00033	0.00033	0.00033
5	NS	NS	NS	NS	–	0.02255	0.03529	0.06732	0.00556	0.00033	0.00425	0.00033	0.00033	0.00033	0.00033	0.00033	0.00033	0.00033
6	NS	NS	NS	*	NS	–	0.25196	0.08137	0.13954	0.00033	0.09510	0.00033	0.00033	0.00033	0.00033	0.00033	0.00556	0.00033
7	*	NS	NS	*	NS	NS	–	0.11928	0.12418	0.00033	0.35752	0.00033	0.00033	0.00033	0.00033	0.00033	0.00163	0.00033
8	NS	NS	NS	NS	NS	NS	NS	–	0.57745	0.06634	0.44739	0.00033	0.00033	0.00033	0.00033	0.00033	0.00817	0.00033
9	NS	NS	NS	NS	NS	NS	NS	NS	–	0.00033	0.05850	0.00033	0.00033	0.00033	0.00033	0.00098	0.03725	0.00033
10	*	*	*	*	*	*	*	NS	*	–	0.04837	0.00033	0.00033	0.00033	0.00033	0.00033	0.00033	0.00033
11	NS	NS	NS	NS	NS	NS	NS	NS	NS	NS	–	0.00033	0.00033	0.00033	0.00033	0.00098	0.27124	0.00033
12	*	*	*	*	*	*	*	*	*	*	*	–	0.00033	0.00033	0.00033	0.00033	0.00033	0.00033
13	*	*	*	*	*	*	*	*	*	*	*	*	–	0.00033	0.00033	0.00033	0.00033	0.00033
14	*	*	*	*	*	*	*	*	*	*	*	*	*	–	0.00033	0.00033	0.00033	0.00033
15	*	*	*	*	*	*	*	*	*	*	*	*	*	*	–	0.00033	0.00033	0.00033
16	*	*	*	*	*	*	*	*	NS	*	NS	*	*	*	*	–	0.55327	0.00033
17	*	*	*	*	NS	NS	NS	NS	NS	*	NS	*	*	*	*	NS	–	0.00033
18	*	*	*	*	*	*	*	*	*	*	*	*	*	*	*	*	*	–

Gray-scale shadding marks different geographic groups. See also Figure 1 for a geographic map of the collection sites and Table 1 for precise location.

Numbers in first column and third row stand for different samples: 1, Agia Larissa; 2, Kallipefki Larissa; 3, Kato Lechonia; 4, Kamari; 5, Karditsa; 6, Pertouli; 7, Konitsa; 8, Dafni1; 9, Dafni2; 10, Kastoria; 11, Salonica; 12, Kernitsa; 13, Chania; 14, Chios; 15, Lesvos; 16, Steckelberg; 17, Dossenheim; 18, Krasnodar.

NS, not significant; *, significant.

### Analysis of the structure of *R. cerasi* natural populations

**Figure 3:**
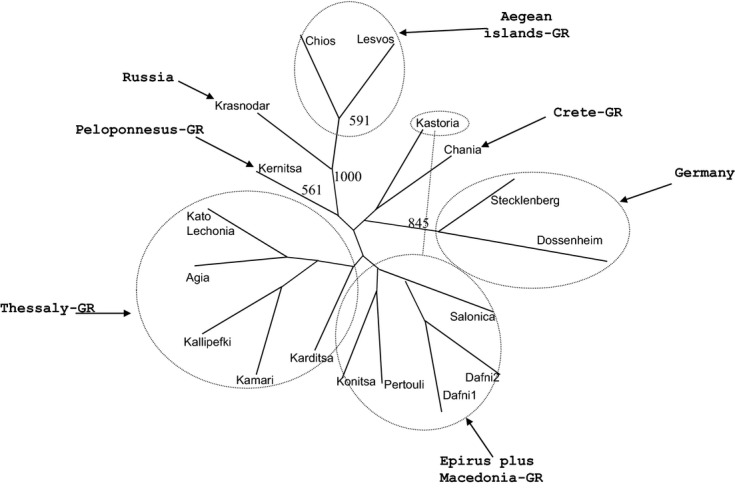
Unrooted UPGMA dendrogram created in Phyllip 3.6c, based on allele frequency differences, after 1000 bootstrap resamples. Only bootstrap values higher than 500 are shown.

**Figure 4:**
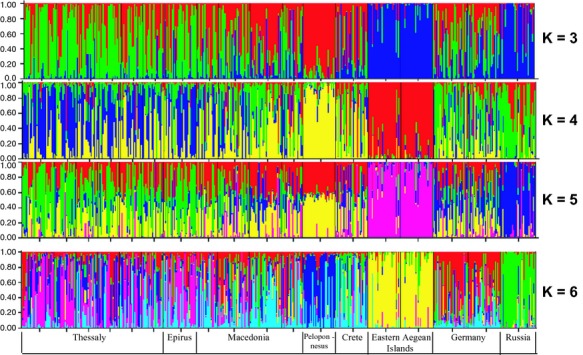
*Rhagoletis cerasi* analysis using Structure, under the assumptions of the existence of three to six genetic groups. See also the curve after the Evanno modification (Figure S2), pointing to the possibility of six genetic clusters.

**Figure 5:**
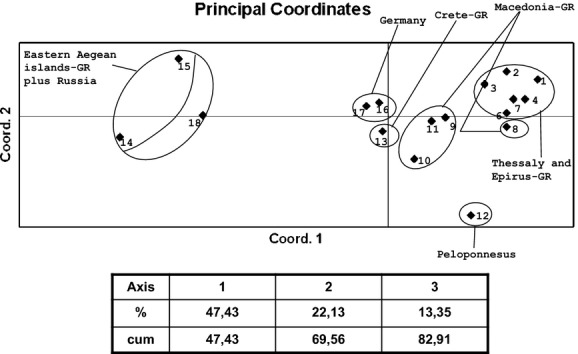
Principal Components Analysis (PCA), using the PhiPT distance matrix created in Genalex 6.5, based on the allele frequency differences among *Rhagoletis cerasi* samples. 1, Agia Larissa; 2, Kallipefki Larissa; 3, Kato Lechonia; 4, Kamari; 5, Karditsa; 6, Pertouli; 7, Konitsa; 8, Dafni1; 9, Dafni2; 10, Kastoria; 11, Salonica; 12, Kernitsa; 13, Chania; 14, Chios; 15, Lesvos; 16, Steckelberg; 17, Dossenheim; 18, Krasnodar.

To further unravel the genetic structure of *R. cerasi* natural populations, different programs and assumptions were used. A UPGMA dendrogram was constructed in Phylip 3.6c (Fig. 3). Bayesian clustering analysis was performed using STRUCTURE 2.2 (Fig. 4), along with the modification in Evanno et al. (2005), which is supposed to give a better resolution of the true number of clusters in complex cases of population structuring. PCA was also performed using Genalex 6.5 (Fig. 5). Although these approaches do not produce exactly the same results, the main conclusion is that there is some structuring with the populations from Krasnodar-RUS and Aegean Sea (Chios-GR and Lesvos-GR) to be strongly differentiated from all others. Differentiation is also evident for Kernitsa-GR (Peloponnesus) and the two German populations. Although there is not strong statistical support, two additional loose clusters may be present which include populations from Thessaly-GR and Macedonia/Epirus-GR.

We used software GeneClass v2.0 to assign individuals to respective groups. Eight groups, based on geographic criteria, were formed: Thessaly-GR, Epirus-GR, Macedonia-GR, Peloponnesus-GR, Crete-GR, Aegean Islands-GR, Germany, and Russia. Some populations are well separated; the vast majority of their individuals are correctly assigned to the respective groups (Fig. 2). This is true for populations from Kernitsa-Peloponnesus (29/30 correctly assigned), the Aegean islands (56/60), Russia (28/32), and Stecklenberg (28/32). There was a high number of false assigned individuals for the remaining populations (ranging from 26% to more than 50% per population), especially for Macedonia-GR, Thessaly-GR, and Dossenheim-GER.

### Isolation by distance

The hypothesis whether the observed genetic distances can be correlated with geographic distances between sampling locations was tested in pairwise comparison of *F*_ST_ values and the logarithm of the geographic distances. There was only a weak correlation (*r* = 0,33, *P* < 0.1) indicating that other factors may also play a significant role in the development of the observed gene flow restriction between *R. cerasi* populations.

### Detection of bottlenecks

The program BOTTLENECK was used for the detection of recent bottlenecks. Results have been handled with care as only few of the populations consist of at least 30 individuals and the loci used in this analysis are <20 and, in addition, they are not highly polymorphic. Given these limitations, no evidence for such phenomena was found with the exception of the Kernitsa population where a shifted mode of allele frequencies from the L- shaped distribution was observed.

### *Wolbachia* infections in *R. cerasi* populations

Using a 16S *rRNA* gene-based PCR screen, all 244 individuals originating from the 18 different *R. cerasi* populations were found infected with *Wolbachia*. To investigate whether there is a correlation between genetic structuring of *R. cerasi* natural populations and the presence of different *Wolbachia* strains, we analyzed six individual flies from different groups as determined by microsatellite analysis and/or based on geographic criteria. These flies derived from the populations of Agia Larissa (Thessaly-GR), Chania (Crete-GR), Chios (Aegean Islands-GR), Kernitsa (Peloponnesus-GR), Krasnodar (Russia), and Stecklenberg (Germany).

PCR products were obtained from these six flies for the 16S *rRNA*, *wsp* as well as the five MLST genes (*coxA, fbpA, ftsZ, gatB,* and *hcpA*). All PCRs produced the expected amplicon and these products were cloned. Two to six clones were sequenced per product and were found to be *Wolbachia* specific: sixteen 16S *rRNA*, twenty-one *coxA*, twenty *fbpA*, twenty-two *ftsZ*, twenty *gatB*, 15 *hcpA,* and eighteen *wsp* clones were sequenced revealing three to five alleles per locus (Table 5)**.** The major findings of the sequencing analysis (Table 5) can be summarized as follows: (a) the majority of the 16S *rRNA* sequences (14/16) form a cluster in supergroup A (Figure S2). There are two exceptions: first, one sequence derived from the fly from Chania clusters in another clade, but still with sequences belonging to supergroup A and second, one sequence derived from the fly from Stecklenberg clusters with sequences belonging to supergroup B; (b) the 21 *coxA* sequences revealed the presence of four alleles (Figure S3). Most of the sequences (16) correspond to allele *coxA-84,* which has been reported in *w*Cer1 and *w*Cer4 strains, and were found in Greek populations (Agia, Chios, Kernitsa) as well as in Krasnodar and in Stecklenberg. The allele *coxA-1*, which is present in *w*Cer2, was detected in Stecklenberg too. Interestingly**,** two new alleles were detected in the individual from Chania-GR. These new alleles are named *coxA-188* and *coxA-189*. *CoxA-188* differs only in 1 bp from allele *coxA-130* and *coxA-189* differs in 1 bp from allele *coxA-58*. However, both cluster near the allele *coxA-84,* which is common in *w*Cer1 and *w*Cer4 strains. None of our *coxA* sequences group with *w*Cer5*,* a *Wolbachia* strain belonging to supergroup B; (c) the 20 *fbpA* sequences suggested the presence of four alleles (*fbpA-1, -4, -79, and -160*) which correspond to alleles characterized so far to four different *w*Cer strains. Most of the clones (15) correspond to allele *fbpA-160*, present in strain *w*Cer1, and originate from Greek populations (Agia, Chania, Chios, Kernitsa) as well as from Stecklenberg and Krasnodar. Two clones correspond to *fbpA-1* of strain *w*Cer2 and were detected in Stecklenberg, two clones correspond to *fbpA-79*, which is assigned to *w*Cer4, and were present in Chania and in Kernitsa while one clone (from Chios-GR) corresponds to *fbpA-4,* which is found in *w*Cer5, the only strain belonging to supergroup B; (d) the 22 *ftsZ* sequences revealed the presence of four alleles (*ftsZ-3, -22, -70,* and *-*79) described for *w*Cer strains. All sequences cluster with *w*Cer strains belonging to supergroup A, with the exception of the sole sequence from Stecklenberg, which clusters with *w*Cer5 of supergroup B (*ftsZ*-22). Thirteen of the clones correspond to the allele *ftsZ*-79 assigned to *w*Cer1 and originate from Greek populations (Agia, Chios, Kernitsa) and from Krasnodar. Six clones correspond to *ftsZ*-70 of *w*Cer4 (originating from Chania, Kernitsa, and Krasnodar) while two clones correspond to *ftsZ*-3 assigned to *w*Cer2; (e) the 20 *gatB* sequences suggested the presence of three *w*Cer alleles belonging to supergroup A (*gatB-1, -8,* and *-53*). Eleven of them correspond to allele *gatB-8* of strain *w*Cer1 originating from Greek populations (Agia, Chios, Kernitsa) as well as from Stecklenberg and Krasnodar. Seven clones are identical to *gatB-53* (*w*Cer4) and were detected in Chania and Krasnodar while two clones are identical to the allele *gatB-1*, which is assigned to strain *w*Cer2, and were found in Stecklenberg; (f) the 15 *hcpA* sequences revealed the presence of five *w*Cer alleles (*hcpA-40, -85, -103, -212,* and -*213*). All of them cluster with *w*Cer sequences of supergroup A, with the exception of two of the three clones from Chios, which are identical to allele *hcpA*-40, which is assigned to *w*Cer5 (Figure S4). Eight clones correspond to allele *hcpA*-103 of strain *w*Cer1 originating from Greek populations (Agia, Chios, Kernitsa) and from Krasnodar. Only two sequences correspond to *hcpA*-85 of *w*Cer4, both detected in Kernitsa. Interestingly, in the individual from Chania, two new alleles were present (*-212, -213*). *HcpA-213* is very similar to *hcpA-103* of strain *w*Cer1 (different in the first 3 bp), while *hcpA-212* is very similar to *hcpA-85* of strain *w*Cer4 (different in the first 3 bp also); and (g) the 18 *wsp* sequences suggested the presence of three known *w*Cer alleles (*wsp-113, -335,* and *-581*). All of them cluster in supergroup A, with the exception of one of the five clones from Chios, which is identical to allele *wsp-581*, which is found in strain *w*Cer5. Fifteen of the sequences were identical to *wsp-335* of *w*Cer1 originating from Greek populations Agia, Kernitsa, Chania, Chios) and from Krasnodar, while two clones from Chania were identical to *wsp-113* of strain *w*Cer4. Taken together these data strongly suggest the presence of multiple *Wolbachia* infections in all *R. cerasi* populations studied.

**Table 5:** MLST and *wsp* alleles found in individual flies derived from *Rhagoletis cerasi* selected populations

	MLST genes										*wsp*		Possible type of infection
	
	*coxA*		*fbpA*		*ftsZ*^*2*^		*gatB*		*hcpA*				
						
	N	allele	N	allele	N	allele	N	allele	N	allele	N	allele	
Agia-GR	3	84 (3)	3	160 (3)	4	79 (4)	4	8 (4)	2	103 (2)	5	335 (5)	*w*Cer 1
Chania-GR	3	**189 (2)** **188(1)**	2	160 (1) 79 (1)	3	70 (3)	4	53 (4)	3	212 (2) 213 (1)	3	113 (2) 335 (1)	*w*Cer 1, 4, other
Chios-GR	4	84 (4)	5	160 (4) 4 (1)	6	79 (4) 3 (2)	4	8 (4)	3	40 (2) 103 (1)	5	335 (4) 581 (1)	*w*Cer 1, 2, 4, 5
Kernitsa-GR	3	84 (3)	3	160 (2) 79 (1)	5	79 (3) 70 (2)	1	8 (1)	4	103 (2) 85 (2)	2	335 (2)	*w*Cer 1, 4
Stecklenberg-GER	3	1 (2) 84 (1)	4	160 (2) 1 (2)	1	22 (1)	3	1 (2) 8 (1)	–	–	–	–	*w*Cer 1, 2, 5
Krasnodar-RUS	5	84 (5)	3	160 (3)	3	79 (2) 70 (1)	4	8 (1) 53 (3)	3	103 (3)	3	335 (3)	*w*Cer 1, 4
*w*Cer1 [A]^1^	84		160		79		8		103		335		
*w*Cer2 [A]^1^	1		1		3		1		1		23		
*w*Cer4 [A]^1^	84		79		70		53		85		113		
*w*Cer5 [B]^1^	5		4		22		101		40		581		

Gray shaded lines: MLST profile of *w*Cer strains, as published by Arthofer et al. (2011).

In bold: new MLST alleles found in this study and deposited in the *Wolbachia* MLST database.

MLST, multi locus sequence typing; N, number of colonies sequenced.

In parentheses (): number of colonies harboring the respective allele.

1 In brackets []: supergroup to which each strain belongs.

The presence of multiple *Wolbachia* infections was also confirmed by a *wsp* gene-based diagnostic PCR assay previously developed (Arthofer et al. 2009b). The primers used in this assay selectively amplify different alleles of the *wsp* gene that have been attributed to the different *w*Cer strains. Ten to twenty individuals were screened per population (Table 6). In accordance with previous studies, all individuals were found infected with *Wolbachia* and all of them harbored at least the *w*Cer1 strain (Riegler and Stauffer 2002; Arthofer et al. 2009b, 2011). The majority of the Greek individuals were found double-infected with *w*Cer1 and *w*Cer4 strains while a few individuals were found triple-infected with different combinations of *w*Cer1, *w*Cer 2, *w*Cer4, and *w*Cer5 strains or infected with four *Wolbachia* strains (*w*Cer1, *w*Cer2, *w*Cer4, and *w*Cer5). It has to be noted, however, that *w*Cer1 single-infected individuals were detected in several populations while one of them, Lesvos-GR, was found to be 100% single-infected with this strain. The majority of Krasnodar-RUS individuals were double-infected with *w*Cer1 and *w*Cer4 strains while triple-infected (*w*Cer1, *w*Cer2 and *w*Cer4) flies were also present. The German *R. cerasi* populations were almost fixed for the presence of four different *Wolbachia* strains: *w*Cer1, *w*Cer2, *w*Cer4, and *w*Cer5. None of the individuals tested was found infected with the *w*Cer3 strain.

**Table 6:** PCR screening of *Rhagoletis cerasi* populations with the primer pairs developed for the detection of *Wolbachia w*Cer 1–5 strains (Arthofer et al. 2009b)

						Type of infection							
						
				*N*	wspec	wcer1	1 + 2	1 + 4	1 + 5	1 + 2 + 4	1 + 2 + 5	1 + 4 + 5	1 + 2 + 4 + 5
1	Agia-GR	Thessaly	Greece	20	20	6		11	1			2	
2	Kallipefki-GR			10	10	8		2					
3	Kato Lechonia-GR			10	10	6		4					
4	Kamari Pilio-GR			10	10			10					
5	Karditsa-GR			10	10			8				2	
6	Pertouli-GR			10	10	3		5				2	
7	Konitsa-GR	Epirus		10	10	5		5					
8	Dafni1-GR	Macedonia		10	10			10				2	
9	Dafni2-GR			10	10			10					
10	Kastoria-GR			10	10			10					
11	Thessaloniki-GR			10	10	4		6					
12	Kernitsa-GR	Peloponnesus		20	20	2		9		1		8	
13	Chania-GR	Crete		19	19	5		9		1		2	2
14	Chios-GR	East Aegean		20	20	7		9				4	
15	Lesvos-GR			14	14	14							
16	Stecklenberg-GER		Germany	20	20								20
17	Dossenheim-GER			10	10					2			8
18	Krasnodar-RUS		Russia	20	20			15		5			

*Wolbachia's* presence was screened with the universal *Wolbachia* 16s *rRNA* primer pairs (wspec F – wspecR).

PCR, polymerase chain reaction; *N*, number of flies screened.Grey shading highlights populations from Germany (light gray) and Russia (dark gray).

## Discussion

*Rhagoletis cerasi* present some unique properties when compared with the medfly and the olive fly. Medfly is a multivoltine and polyphagous species (Papadopoulos et al. 2001), the olive fly is a multivoltine and monophagous species (Tzanakakis 2003), while the cherry fruit fly is a univoltine and stenophagous species (Moraiti et al. 2012a,b2012b). These three species also differ in their *Wolbachia* infection status: natural populations of olive fly and medfly are *Wolbachia* free (Bourtzis et al. 1994; Zabalou et al. 2004; Apostolaki et al. 2011; but see Rocha et al. 2005 for an infected Brazilian population) while *Wolbachia* has been detected in all natural populations of the European cherry fruit fly tested so far. Our results indicate the presence of structuring in natural populations of *R. cerasi*, which, especially for Greece, is not in accordance with data for the olive fly (Augustinos et al. 2005; Zygouridis et al. 2009)*,* and the medfly (K. Economou et al. unpubl. data), which point to rather homogeneous groups for both. Is then the genetic structuring observed in natural cherry fruit fly populations due to ecological factors or rather due to the presence of different/multiple *Wolbachia* strains?

### Variability of microsatellite markers and HWE

We used 13 microsatellite markers; 10 cross-amplified from other *Rhagoletis* species (see Augustinos et al. 2011a), one from the olive fly (Augustinos et al. 2008), and two of the set that has been developed de novo for *R. cerasi* (Arthofer et al. 2009a). Some of the cross-amplified markers identified low polymorphism compared with species in which they were developed. This is not surprising as it is known that cross-amplification of microsatellite markers includes three major risks: (a) amplification of nonhomologous regions, (b) amplification of homologous regions with fewer or disrupted repeats, leading therefore to lower mutation rates, and (c) poor amplification in some or most of the individuals, due to changes in primer binding sites. Sequencing analysis of cross-amplified products verified that we amplified homologous regions (data not shown), with the exception of RcMic (Boms3b) from the olive fly and RcRp1 from *R. pomonella*. In most of the cases, microsatellites with fewer and/or interrupted repeats or with new motifs (data not shown) were present, explaining the reduced polymorphism. There was also evidence of size variation not attributed to motif changes, even within *R. cerasi* (data not shown), implying the participation of other events, such as unequal crossing over and/or indels affecting the microsatellite marker size. Although 13 de novo developed microsatellite markers were available for *R. cerasi* (Arthofer et al. 2009a)*,* we managed to use only two of them. In preliminary trials, we found that scoring of the remaining 11 markers was very difficult and unreliable, also due to secondary bands and poor quality of main bands (faint bands or not sharp enough to be scored). This is probably because these markers harbor multiple motifs, one of which being one base pair repeat motif, making their analysis problematic, especially in polyacrylamide gels.

Deviations from HWE were found in few of the populations for some of the cross-amplified loci but not for the two species-specific microsatellite markers (Table 2). This could partially be attributed to the small sample size (in some cases) or the presence of null alleles, as mentioned above. Evidence for a relative extended presence of null alleles in some of the samples was found only for *Rcmic(Rp11)*.

### *Rhagoletis cerasi* natural populations' polymorphism

All populations exhibited a comparable degree of polymorphism. The most polymorphic population is the one from Krasnodar-RUS. This can be explained considering that *R. cerasi* moved to Europe from western Asia through the Caucasus. This population should therefore be closest to the original expansion area of the species. Populations near the expansion center of a species are expected to be more polymorphic, if no drastic bottlenecks have occurred and populations have maintained a relative large size through time. On the other hand, populations from the Eastern Aegean islands (Chios and Lesvos) along with Kernitsa from Peloponnesus present the lowest polymorphism. This can be attributed to an island effect, as isolated populations are expected to be less polymorphic, due to restriction of gene flow and probably small effective population size. As far as the sample from Kernitsa (Peloponnesus) is concerned, Peloponnesus is not a true island and communicates with the rest of mainland Greece; however, cherry orchards in the area are few and dispersed. Another factor to be taken into account is that the sample from Kernitsa consists of individuals that have undergone prolonged dormancy, while all other samples consist of flies that emerged after annual dormancy. Although the prolonged dormancy in *R. cerasi* pupae seems to be a plastic response to environmental cues (C. A. Moraiti and N. T. Papadopoulos unpubl. data), its possible impact on population structuring including bottleneck phenomena deserves further investigation.

### Structuring of populations

Despite the low overall polymorphism observed, the markers used here identified well and poorly separated clusters (Figs. 2, 3, 4, 5). The well separated groups included populations from Aegean islands-GR and Krasnodar-RUS followed by Kernitsa-GR and German samples. The two loose groups were those from Macedonia/Epirus-GR and Thessaly-GR. Despite its geographical isolation, the single population from Chania was not well differentiated probably due to trade and airport connections between Crete and many mainland regions in Greece and other European countries including Germany.

The genetic difference of the samples collected from islands or “island”-like sites (Lesvos, Chios, Kernitsa) is accompanied by a slightly lower degree of polymorphism (Table 1). This is in accordance with the hypothesis of partially isolated populations. No evidence of recent bottlenecks was found (with the exception of Kernitsa population discussed above) suggesting that we are probably dealing with long-standing populations that manage to keep a relative high population density through time.

Another finding, although with low support (Figs. 3, 5), is the topology of the Macedonian-GR group of samples (especially the sample from Kastoria): as said before, they form a loose group, placed near the Thessaly-GR cluster, having, however, a tendency to link with the German cluster. If we take into account that Thessaly is in Central Greece and Macedonia-GR is mainly in Northern Greece and is the region that neighbors with Albania, Former Yugoslav Republic of Macedonia and Bulgaria, we can speculate in favor of the existence of gene flow from Northern Europe in this area. The lack of samples from this area limits our analysis.

Samples from the two Eastern Aegean islands are strongly differentiated from all other Greek and German samples. They seem to form a “loose” cluster with the sample from Russia, pointing toward gene flow from Asia Minor, most probably through Turkey, a major cherry producer. Analysis of samples from the coastal area of Turkey all the way to the coast of Black Sea would definitely shed light on gene flow from Asian to European *R. cerasi* populations. It is worth noting that these island populations do not form one panmictic population, despite the fact that the distance between them is only 90 km.

Despite differences in genetic distance within the same geographic scale between *R. cerasi, C. capitata,* and *B. oleae,* the degree of polymorphism was comparable or even smaller in *R. cerasi* (though the same markers couldn't be used). Therefore, these differences are not marker biased. They are also not population biased, as relatively large samples were used in all studies (20–30 individuals per population). It seems that the fragmented landscape of mainland Greece, together with a patchy distribution of cherry orchards, the low dispersion ability of the cherry fly and local adaptation from close synchronization of adult flight with the sweet cherry ripening period may restrict gene flow to relatively short distances (Phillips and Dirks 1933; Jones and Wallace 1955; Boller and Prokopy 1976; Fletcher 1989; Kneifl et al. 1997). Indeed, recent evidence demonstrates high differences in diapause intensity between coastal and highland *R. cerasi* populations (Papanastasiou et al. 2011) and extended difference in adult life history traits between populations (Moraiti et al. 2012b). Low reproduction rates of *R. cerasi* because of univoltinism and stenophagy may also partially explain differences in genetic structure against both *C. capitata* and *B. oleae*. On the other hand, infection of all natural populations of *R. cerasi* with different strains of *Wolbachia* may be yet another factor contributing toward structuring and genetic isolation.

### *Wolbachia* infections in *R. cerasi*

*Wolbachia* is assumed to invade natural populations rapidly and is implicated in speciation phenomena and restriction of gene flow between natural populations (Shoemaker et al. 1999; Bordenstein et al. 2001; Jaenike et al. 2006; Koukou et al. 2006; Miller et al. 2010; Branca et al. 2011). This can be either through CI and/or rapid fixation of specific genotypes during invasion. Natural European *R. cerasi* populations are infected with multiple *Wolbachia* strains and show different infection patterns. For example, in Poland, Italy, and Austria, there are populations infected with all five *w*Cer strains, in the Czech Republic and Portugal there are populations infected with four of the five strains (except *w*Cer2), while in Switzerland there was an infection with all *w*Cer strains except *w*Cer3 (Arthofer et al. 2009b, 2011). Moreover, it has been shown that the presence of different *Wolbachia* strains in *R. cerasi* could be the causal factor (Riegler and Stauffer 2002) of the incompatibility observed among *R. cerasi* natural populations in previous studies (Boller et al. 1976; Matolin 1976).

All individuals in this study (10–20 per population) were infected with *Wolbachia*. The analysis of six individuals from different samples (genetically or geographically distant) through cloning and sequencing analysis, as well as the PCR screening of more individuals with the diagnostic PCR primers, pointed toward (a) the presence of complex patterns of infections with the four of the five known *w*Cer strains (1, 2, 4, and 5) and (b) the possible existence of new, still uncharacterized strains (see Chania population). However, not all genes resulted into the same infection pattern (Tables 5, 6). This can be attributed to the preferential recovery of specific sequences after PCR and/or cloning. Overall, these two approaches (PCR screening and cloning plus sequencing) produced similar results and supported the presence of multiple infections. However, both PCR and cloning can lead in bias in favor of specific sequences and to the misinterpretation of the “actual” infection status. On the other hand, PCR screening allows the analysis at population level but currently is limited by the development of strain-specific primer pairs only for the *wsp* gene and by the inability to detect “hidden” strains.

Arthofer et al. (2011) highlighted the difficulties in *Wolbachia* strain characterization through MLST in multiple-infected hosts. They also provided a tool to analyze complex infection types (Allelic Intersection Analysis) and evaluated this tool for *w*Cer *Wolbachia* strains of *R. cerasi* by assigning specific MLST alleles to four of the five *w*Cer strains (Table 5). It is evident that the MLST- and *wsp*-based typing approaches do not have the power to resolve complex infection patterns such as those observed in *R. cerasi*. There is probably the need for new tools and approaches to address such questions. Current use and evaluation of different types of molecular markers, including Variable Number Tandem Repeat markers (VNTRs) (Riegler et al. 2005, 2012; Schneider et al. 2013) can probably contribute toward this direction.

Multiple and/or low titer strains have been shown to contribute to genetic differentiation of their hosts (Arthofer et al. 2009b, 2011; Schneider et al. 2013). Our data confirm the presence of multiple infections, different types of infections in different samples and polymorphic infection status within the samples. They also raise the possibility of the presence of still unknown strains. Given also the ability of at least two strains (*w*Cer2 and *w*Cer4) to induce CI (Boller et al. 1976; Riegler et al. 2004; Zabalou et al. 2004, 2009; Apostolaki et al. 2011), *Wolbachia* is not a factor that can be overlooked when addressing the question of population structuring in *R. cerasi*.

Recombination has perhaps been the most significant contributing factor to the evolution of *Wolbachia* and may, at least partly, explain the diversity of the *w*Cer strains present in European populations of *R. cerasi* (Jiggins 2002; Baldo and Werren 2007; Arthofer et al. 2009b; Klasson et al. 2009). Low titer infections are an additional, hidden source of *Wolbachia* diversity, as documented in different hosts (Arthofer et al. 2009b; Miller et al. 2010; Augustinos et al. 2011b). There is also a number of studies showing that the transfer of *Wolbachia* genes (in some cases even the entire genome) into host chromosomes might be a rather common phenomenon (Kondo et al. 2002; Hotopp et al. 2007; Nikoh et al. 2008; Aikawa et al. 2009; Klasson et al. 2009; Nikoh and Nakabachi 2009; Woolfit et al. 2009; Doudoumis et al. 2012). If this is the case, then nucleus-encoded *Wolbachia* genes may also contribute to the observed genetic diversity. However, our data do not provide evidence for either (intragene) recombination or horizontal gene transfer events.

*Do all wCer Wolbachia strains induce CI?* The fact that *R. cerasi* is a univoltine species and an efficient and robust lab rearing system is lacking makes any experimentation to address questions regarding the CI properties of *w*Cer strains very difficult. Interpretation of historical data in European populations of *R. cerasi* as well as transinfection experiments with medfly and olivefly suggest that *w*Cer2 and *w*Cer4 induce strong CI into their hosts and are bidirectionally incompatible (Boller et al. 1976; Riegler and Stauffer 2002; Riegler et al. 2004; Zabalou et al. 2004, 2009; Apostolaki et al. 2011). On the other hand, *Drosophila simulans* was not shown to be a suitable host for the establishment and/or the expression of CI for either *w*Cer1 or *w*Cer2 strains (Riegler et al. 2004). Thus, transinfection experiments in suitable hosts like medfly can probably unravel the CI properties of *w*Cer3, wCer5, and the two potentially new *w*Cer strains.

## Concluding Remarks

Our results reveal some structuring of natural European cherry fruit fly populations, which can probably be generalized to its whole geographic range. A number of factors may have contributed to this structuring, such as habitat fragmentation as well as *Wolbachia* infection. Microsatellite analysis of more populations obtained from geographically distant areas, along with further genetic characterization of *Wolbachia* strains and their CI properties are required to understand the population dynamics of this economically important species and its associated *Wolbachia* strains more completely. This knowledge will be needed before any novel population control method is applied on a large scale for this important pest species.

## Supporting Information

**Figure S1.** Posterior probability of possible number of populations in STRUCTURE. Note the potential presence of six genetic clusters indicated by the arrow in diagram (D).

**Figure S2.** Maximum Likelihood dendrogram based on the 16S *rRNA* gene sequences. Gray shade: sequences generated in this study. *Wolbachia* 16S *rRNA* gene sequences representing current known Supergroups were retrieved from Genebank. For these sequences, the host scientific name, the Genebank Accession Number and the respective supergroup are shown. Analyses were conducted in Mega 5 (Tamura et al. 2011). The evolutionary history was inferred using the Maximum Likelihood method based on the Tamura–Nei model. The tree with the highest log likelihood is shown and percentages of trees in which the associated taxa clustered together are indicated next to the branches.

**Figure S3.** Maximum Likelihood dendrogram based on the *coxA* gene sequences. Gray shade: sequences generated in this study. Darker gray shade and bold: sequences of known *w*Cer strains. Different *Wolbachia coxA* alleles were retrieved from the *Wolbachia*MLST database. Alleles representing all available supergroups were chosen. New alleles *coxA*-188 and *coxA*-189 are marked with asterisks (*). Analyses were conducted in Mega 5 (Tamura et al. 2011). The evolutionary history was inferred using the Maximum Likelihood method based on the Tamura–Nei model. The tree with the highest log likelihood is shown and percentages of trees in which the associated taxa clustered together are indicated next to the branches.

**Figure S4.** Maximum Likelihood dendrogram based on *hcpA* gene sequences. Gray shade: sequences generated in this study. Darker gray shade and bold: sequences of known *w*Cer strains. Different *Wolbachia hcpA* alleles were retrieved from the *Wolbachia*MLST database. Alleles representing all available supergroups were chosen. New alleles hpA-212 and hcpA-213 are marked with asterisks (*). Analyses were conducted in Mega 5 (Tamura et al. 2011). The evolutionary history was inferred using the Maximum Likelihood method based on the Tamura–Nei model. The tree with the highest log likelihood is shown and percentages of trees in which the associated taxa clustered together are shown next to the branches.
