# Supplementary material for: Microsatellite and Wolbachia analysis in Rhagoletis cerasi natural populations: population structuring and multiple infections
Source: Ecol Evol. 2014 Apr 21;4(10):1943–62. doi: 10.1002/ece3.553 (PMC4063487; doi:10.1002/ece3.553)
Supplement: Supplementary file 1 [file ece30004-1943-SD1.pptx]

## Slide 1
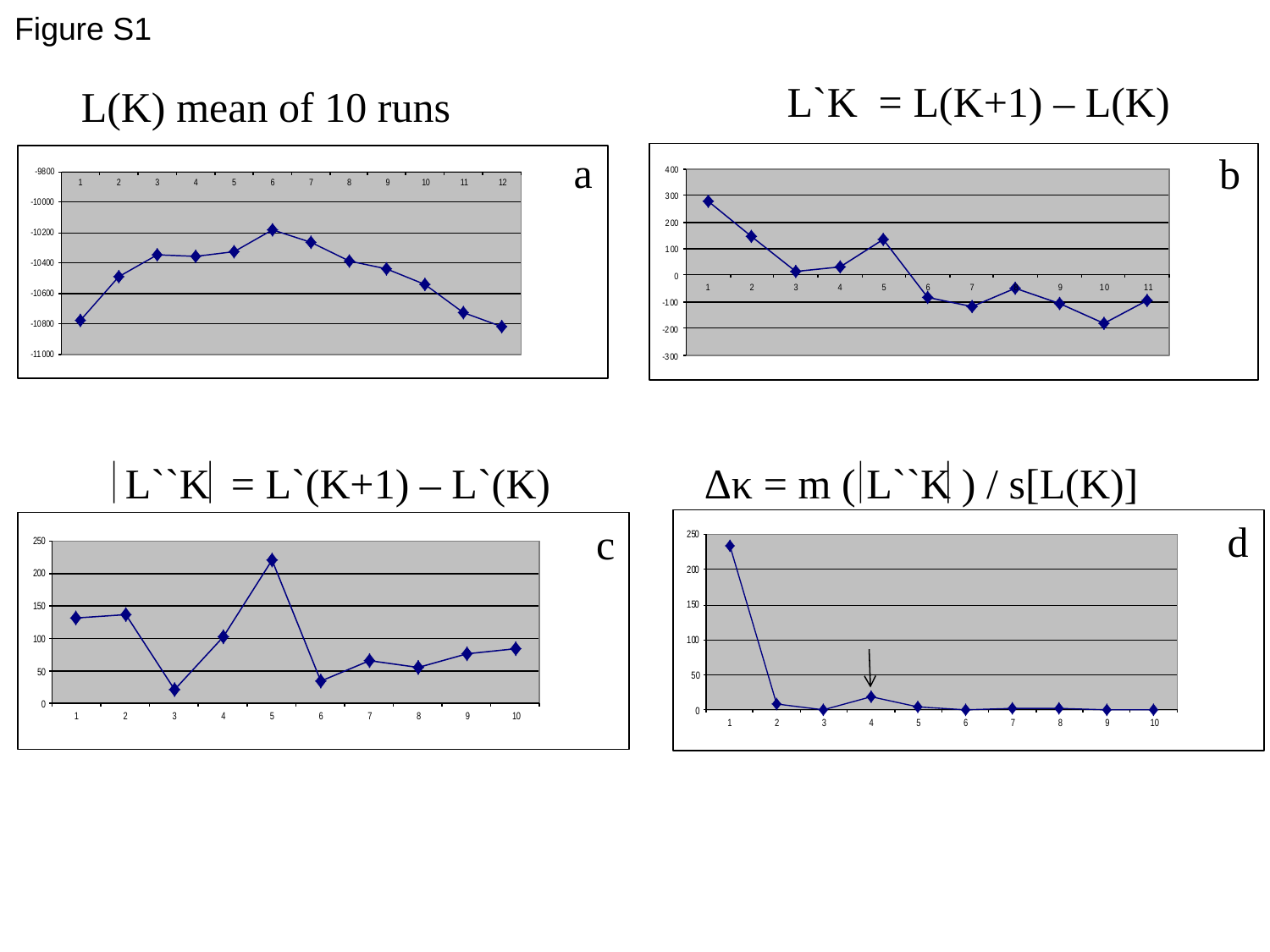

Figure S1
L`K = L(K+1) – L(K)
L(K) mean of 10 runs
a
b
L``K = L`(K+1) – L`(K)
Δκ = m ( L``K ) / s[L(K)]
d
c

## Slide 2
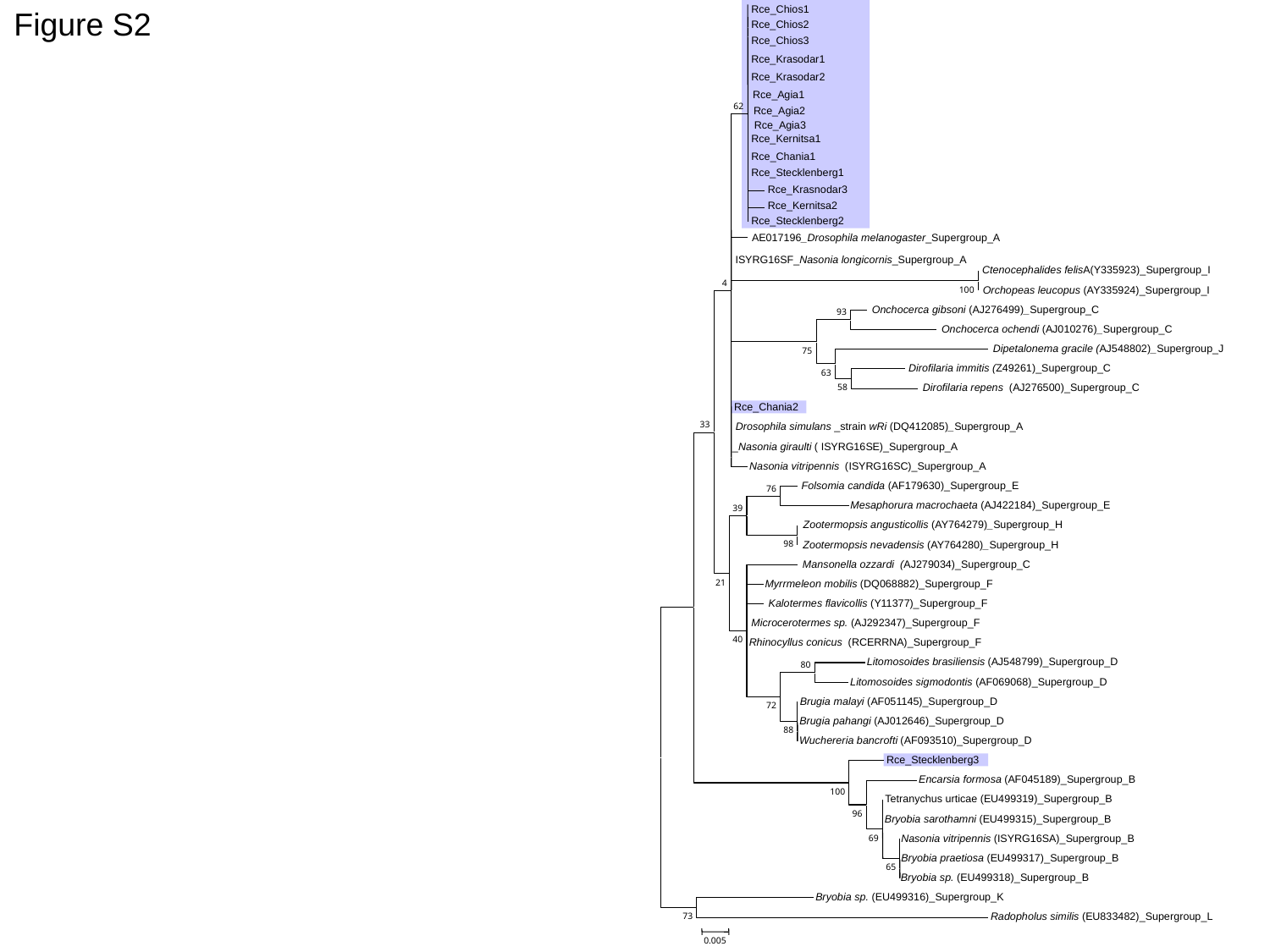

Rce_Chios1
 Rce_Chios2
 Rce_Chios3
 Rce_Krasodar1
 Rce_Krasodar2
 Rce_Agia1
62
 Rce_Agia2
 Rce_Agia3
 Rce_Kernitsa1
 Rce_Chania1
 Rce_Stecklenberg1
 Rce_Krasnodar3
 Rce_Kernitsa2
 Rce_Stecklenberg2
 AE017196_Drosophila melanogaster_Supergroup_A
 ISYRG16SF_Nasonia longicornis_Supergroup_A
 Ctenocephalides felisA(Y335923)_Supergroup_I
4
 Orchopeas leucopus (AY335924)_Supergroup_I
100
 Onchocerca gibsoni (AJ276499)_Supergroup_C
93
 Onchocerca ochendi (AJ010276)_Supergroup_C
 Dipetalonema gracile (AJ548802)_Supergroup_J
75
 Dirofilaria immitis (Z49261)_Supergroup_C
63
 Dirofilaria repens (AJ276500)_Supergroup_C
58
 Rce_Chania2
33
 Drosophila simulans _strain wRi (DQ412085)_Supergroup_A
_Nasonia giraulti ( ISYRG16SE)_Supergroup_A
Nasonia vitripennis (ISYRG16SC)_Supergroup_A
Folsomia candida (AF179630)_Supergroup_E
76
Mesaphorura macrochaeta (AJ422184)_Supergroup_E
39
Zootermopsis angusticollis (AY764279)_Supergroup_H
Zootermopsis nevadensis (AY764280)_Supergroup_H
98
Mansonella ozzardi (AJ279034)_Supergroup_C
21
Myrrmeleon mobilis (DQ068882)_Supergroup_F
Kalotermes flavicollis (Y11377)_Supergroup_F
Microcerotermes sp. (AJ292347)_Supergroup_F
40
Rhinocyllus conicus (RCERRNA)_Supergroup_F
Litomosoides brasiliensis (AJ548799)_Supergroup_D
80
Litomosoides sigmodontis (AF069068)_Supergroup_D
Brugia malayi (AF051145)_Supergroup_D
72
Brugia pahangi (AJ012646)_Supergroup_D
88
Wuchereria bancrofti (AF093510)_Supergroup_D
 Rce_Stecklenberg3
Encarsia formosa (AF045189)_Supergroup_B
100
Tetranychus urticae (EU499319)_Supergroup_B
96
Bryobia sarothamni (EU499315)_Supergroup_B
Nasonia vitripennis (ISYRG16SA)_Supergroup_B
69
Bryobia praetiosa (EU499317)_Supergroup_B
65
Bryobia sp. (EU499318)_Supergroup_B
Bryobia sp. (EU499316)_Supergroup_K
Radopholus similis (EU833482)_Supergroup_L
73
0.005
Figure S2

## Slide 3
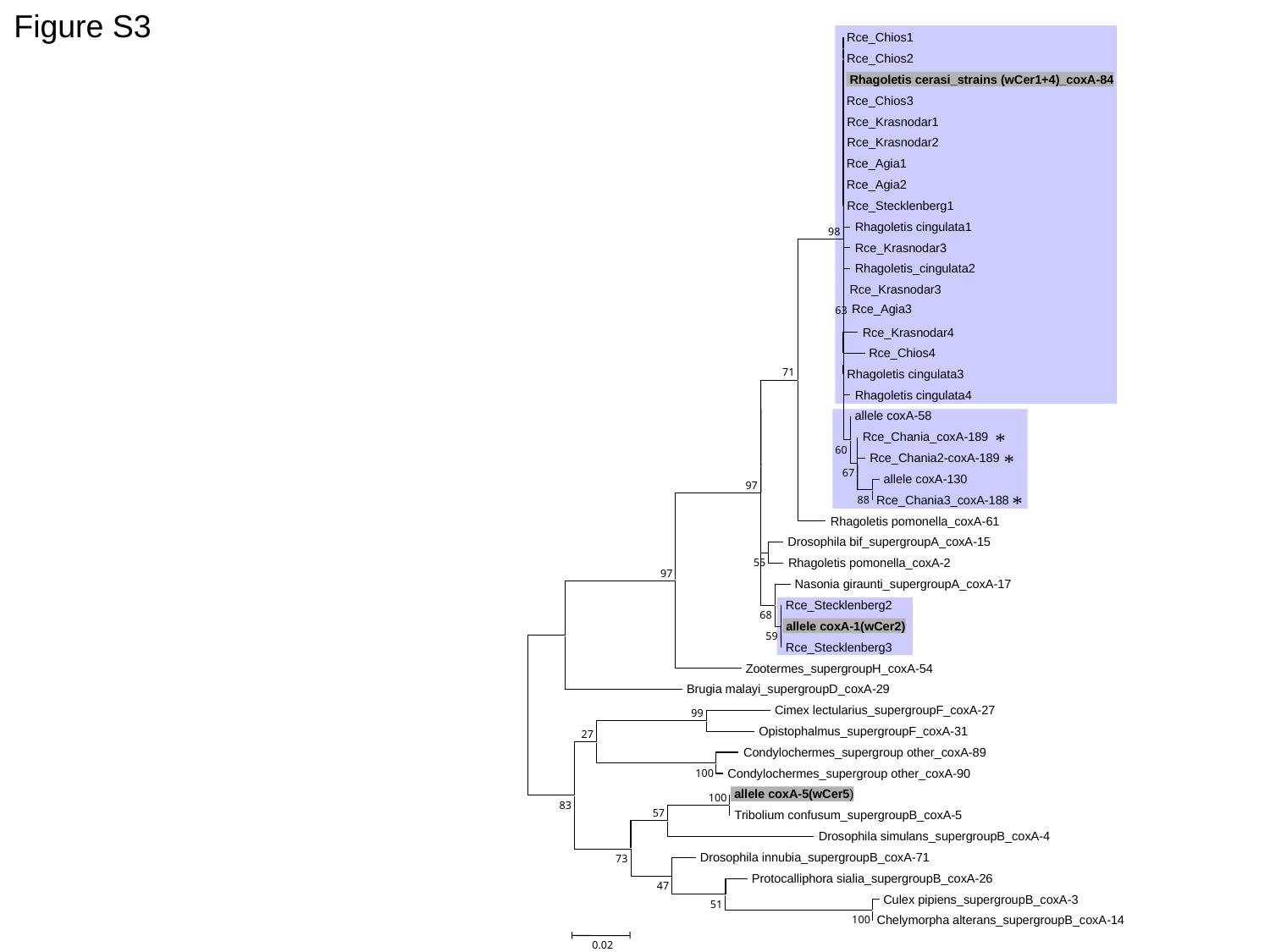

Figure S3
 Rce_Chios1
 Rce_Chios2
 Rhagoletis cerasi_strains (wCer1+4)_coxA-84
 Rce_Chios3
 Rce_Krasnodar1
 Rce_Krasnodar2
 Rce_Agia1
 Rce_Agia2
 Rce_Stecklenberg1
 Rhagoletis cingulata1
98
 Rce_Krasnodar3
 Rhagoletis_cingulata2
 Rce_Krasnodar3
 Rce_Agia3
63
 Rce_Krasnodar4
 Rce_Chios4
71
 Rhagoletis cingulata3
 Rhagoletis cingulata4
 allele coxA-58
*
 Rce_Chania_coxA-189
*
60
 Rce_Chania2-coxA-189
67
 allele coxA-130
97
*
 Rce_Chania3_coxA-188
88
 Rhagoletis pomonella_coxA-61
 Drosophila bif_supergroupA_coxA-15
 Rhagoletis pomonella_coxA-2
55
97
 Nasonia giraunti_supergroupA_coxA-17
 Rce_Stecklenberg2
68
 allele coxA-1(wCer2)
59
 Rce_Stecklenberg3
 Zootermes_supergroupH_coxA-54
 Brugia malayi_supergroupD_coxA-29
 Cimex lectularius_supergroupF_coxA-27
99
 Opistophalmus_supergroupF_coxA-31
27
 Condylochermes_supergroup other_coxA-89
 Condylochermes_supergroup other_coxA-90
100
 allele coxA-5(wCer5)
100
83
57
 Tribolium confusum_supergroupB_coxA-5
 Drosophila simulans_supergroupB_coxA-4
 Drosophila innubia_supergroupB_coxA-71
73
 Protocalliphora sialia_supergroupB_coxA-26
47
 Culex pipiens_supergroupB_coxA-3
51
 Chelymorpha alterans_supergroupB_coxA-14
100
0.02

## Slide 4
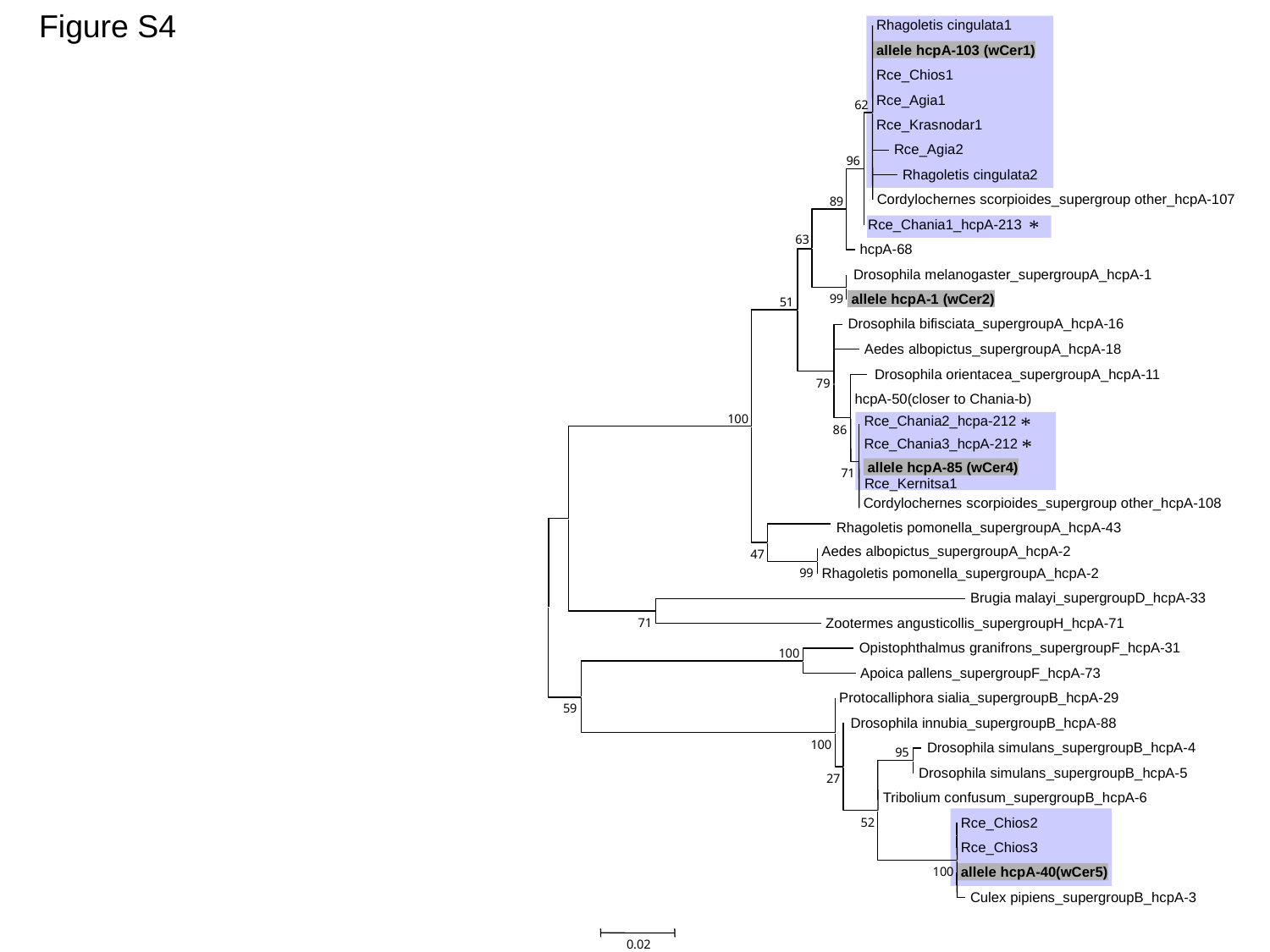

Figure S4
 Rhagoletis cingulata1
 allele hcpA-103 (wCer1)
 Rce_Chios1
 Rce_Agia1
62
 Rce_Krasnodar1
 Rce_Agia2
96
 Rhagoletis cingulata2
 Cordylochernes scorpioides_supergroup other_hcpA-107
89
*
 Rce_Chania1_hcpA-213
63
 hcpA-68
 Drosophila melanogaster_supergroupA_hcpA-1
 allele hcpA-1 (wCer2)
99
51
 Drosophila bifisciata_supergroupA_hcpA-16
 Aedes albopictus_supergroupA_hcpA-18
 Drosophila orientacea_supergroupA_hcpA-11
79
 hcpA-50(closer to Chania-b)
*
100
 Rce_Chania2_hcpa-212
86
*
 Rce_Chania3_hcpA-212
 allele hcpA-85 (wCer4)
71
 Rce_Kernitsa1
 Cordylochernes scorpioides_supergroup other_hcpA-108
 Rhagoletis pomonella_supergroupA_hcpA-43
 Aedes albopictus_supergroupA_hcpA-2
47
 Rhagoletis pomonella_supergroupA_hcpA-2
99
 Brugia malayi_supergroupD_hcpA-33
 Zootermes angusticollis_supergroupH_hcpA-71
71
 Opistophthalmus granifrons_supergroupF_hcpA-31
100
 Apoica pallens_supergroupF_hcpA-73
 Protocalliphora sialia_supergroupB_hcpA-29
59
 Drosophila innubia_supergroupB_hcpA-88
100
 Drosophila simulans_supergroupB_hcpA-4
95
 Drosophila simulans_supergroupB_hcpA-5
27
 Tribolium confusum_supergroupB_hcpA-6
 Rce_Chios2
52
 Rce_Chios3
 allele hcpA-40(wCer5)
100
 Culex pipiens_supergroupB_hcpA-3
0.02
